# Natural Killer (NK) Cell-Based Therapies Have the Potential to Treat Ovarian Cancer Effectively by Targeting Diverse Tumor Populations and Reducing the Risk of Recurrence

**DOI:** 10.3390/cancers17233862

**Published:** 2025-12-01

**Authors:** Kawaljit Kaur

**Affiliations:** ImmuneLink, LLC, Riverside, CA 92508, USA; drkawalmann@g.ucla.edu

**Keywords:** ovarian cancer, NK cells, chemotherapy, cancer stem cells, differentiation, cytotoxicity, IFN-γ, TNF-α

## Abstract

Ovarian cancer is the sixth leading cause of cancer-related deaths among women in the United States. This complex disease arises from tissues such as the ovarian surface epithelium, fallopian tube epithelium, endometrium, or ectopic Müllerian components and is characterized by diverse histological and molecular traits. Standard treatments like surgery, chemotherapy, and radiation have limited effectiveness and high toxicity. Targeted therapies, including poly (ADP-ribose) polymerase PARP inhibitors, anti-angiogenics, and immune checkpoint inhibitors (ICIs), face obstacles such as adaptive resistance and microenvironmental barriers that affect drug delivery and immune responses. Factors in the tumor microenvironment, such as dense stroma, hypoxia, immune suppression, cancer stem cells (CSCs), and angiogenesis, can reduce drug efficacy, worsen prognosis, and increase the risk of recurrence. Research highlights impaired immune function in ovarian cancer patients as a contributor to recurrence, emphasizing the importance of immunotherapies to target tumors and restore immune function. Preclinical studies and early clinical trials found that natural killer (NK) cell-based therapies have great potential to tackle ovarian tumors. This review explores the challenges and opportunities in treating ovarian cancer, focusing on how NK cells could help overcome these obstacles. Recent findings reveal that engineered NK cells, unlike their primary NK cells, can destroy both stem-like and differentiated ovarian tumors, pointing to their ability to target diverse tumor types. Animal studies on NK cell therapies for solid cancers have shown smaller tumor sizes, tumor differentiation in vivo, recruitment of NK and T cells in the tumor environment and peripheral tissues, restored immune function, and fewer tumor-related systemic effects—suggesting a lower chance of recurrence. NK cells clinical trials in ovarian cancer patients have also shown encouraging results, and future directions include combining NK cell therapies with standard treatments to potentially boost effectiveness.

## Simple Summary

This report explores why current ovarian cancer treatments often fall short, pointing to challenges like late detection, impaired immune function, an immunosuppressive tumor environment, and tumor diversity. Natural killer (NK) cells could address these issues by targeting a range of tumor types and lowering recurrence. Studies suggest boosting NK cell-based therapies could improve results, especially alongside existing treatments. Unlike primary NK cells, engineered NK cells can kill both stem-like and mature ovarian tumors, making them effective against diverse tumor populations. Animal research shows NK therapies can shrink tumors, encourage tumor differentiation, draw NK and T cells to tumors and nearby tissues, restore immune function, and reduce tumor-related side effects—potentially decreasing recurrence risk. Early clinical trial results are promising, and future efforts may focus on pairing NK cell therapies with standard care to enhance outcomes while tackling their remaining challenges.

## 1. Introduction

Ovarian cancer is the most lethal gynecologic cancer in the Western world and a major cause of cancer deaths in the United States [1,2]. Advanced ovarian cancer has a five-year survival rate of less than 30%, while early-stage cases (stage I) can have a cure rate of up to 90% [3]. Unfortunately, early detection is tough due to vague symptoms, and over 70% of cases are diagnosed at stage III or IV, where survival rates drop below 50% [3,4]. Risk factors include genetic mutations like *BRCA1/2*, *Lynch syndrome*, *TP53* variants, hormonal and reproductive factors, endometriosis, obesity, diet, and possibly talc exposure [5]. Frequent ovulation can damage deoxyribonucleic acid (DNA), causing cancerous cysts, while oxidative stress and inflammation from ovulation can increase mutation rates [2]. Risk rises significantly after age 55 due to genomic instability, telomere shortening, cell aging, reduced DNA repair, and weakened immunity [3,4]. Treatment usually involves surgery and chemotherapy, with radiation and immunotherapy used in some cases, though effectiveness is limited [5] or is associated with severe toxicities or adverse effects [6,7]. Thus, the challenges of managing ovarian cancer include late detection, microscopic spread, tumor diversity, adaptive resistance, short treatment responses, and an immunosuppressive tumor environment [8].

Interest in cancer immunotherapies is exploding, most explored including chimeric antigen receptor (CAR) T lymphocyte, monoclonal antibodies, checkpoint inhibitors, bispecific antibodies, T cell redirecting antibodies, vaccination strategies, T-cell receptor (TCR) gene-modified T cells, donor lymphocyte infusions (DLI), and antigen-specific DLI [6,9,10]. However, limitations using T cells and autologous cell products are apparent as they take weeks to produce, rely on a one-to-one donor-to-patient model, are costly, and are susceptible to variability and manufacturing failures [11]. CAR T cells are also associated with significant toxicities, including cytokine release syndrome, immune effector cell–associated neurotoxicity syndrome, and prolonged cytopenia [11]. To overcome these issues, natural killer (NK) cells are being explored as an alternative cell source for allogeneic cell therapies and are currently being investigated in ongoing clinical trials as a single therapy or combination therapy, and have thus far yielded many encouraging clinical results [11].

NK cells are innate immune cells, representing approximately 5 to 20% of total lymphocytes in human peripheral blood, and are known for their anticancer properties [12,13]. They originate from common lymphoid progenitors in the bone marrow and further diversify in peripheral tissues like the spleen, liver, and lymph nodes, adapting to their environment [14,15,16]. Unlike other innate lymphoid cells (ILCs), they have distinct transcriptional programs that drive their cytotoxic functions. Key surface markers such as CD56, CD16, NKG2D, NKp30, NKp44, and NKp46 help them detect infected or tumor cells, balance activation and inhibition, and regulate cytokine release and killing activity [12,13]. CD56 distinguishes NK cell subsets: CD56^bright^ cells excel at producing cytokines, while CD56^dim^ cells are strong cytotoxic effectors [17]. CD16 (FcγRIIIa) mediates antibody-dependent cellular cytotoxicity (ADCC) by binding IgG-coated targets [18]. NKG2D activates NK cells by recognizing stress-induced ligands on abnormal cells [19]. NKp30, NKp44, and NKp46 are natural cytotoxicity receptors that trigger direct target killing [20]. Inhibitory receptors like killer-cell immunoglobulin-like receptors (KIRs) detect MHC class I to protect healthy “self” cells, while CD94/NKG2A helps maintain tolerance to normal cells [14]. NK cells can directly recognize and destroy tumor cells without prior sensitization, while also coordinating with adaptive immune responses [21]. Their antitumor functions include direct cytotoxicity, ADCC, and the indirect influence of other immune effectors by releasing inflammatory cytokines and chemokines [13,22]. Preclinical studies have shown that conventional NK cells can target ovarian cancer stem-like cells (CSCs) [23], and engineered NK cells lyse both CSCs as well as differentiated ovarian cancers [24]. Preclinical and early phase clinical trials have demonstrated efficacy and persistence of NK cells against ovarian cancer [25].

This report explores impaired immune function in the peripheral blood as well as in the tumor microenvironment of ovarian cancer patients, tumor heterogeneity in ovarian cancer, and how NK cells can overcome these challenges, targeting the complex tumor environment while also reducing the risk of recurrence. Current ovarian therapeutics and factors contributing to reduced therapeutic benefits have been reviewed. It covers the hurdles in developing NK cell-based therapies and highlights progress made to boost their success. The report provides an in-depth review of preclinical and clinical approaches of NK cell-based therapies for ovarian cancer. While preclinical research shows NK cells have promise, issues like their persistence and survival in the body still limit their use for patients. To address this, engineered or memory-like NK cells are being explored and have shown encouraging results in early clinical trials. Lastly, it proposes ideas for improving ovarian cancer treatment strategies in the future.

## 2. Factors Contributing to Late-Stage Detection of Ovarian Cancer

Ovarian cancer is hard to detect early, which contributes to its high death rate [8]. Challenges include biological, clinical, diagnostic, and systemic factors [8]. Unlike breast or cervical cancer, ovarian cancer still does not have reliable, standardized screening programs [26]. While identifying molecular subtypes and genetic mutations like *BRCA1/2* and *TP53* has advanced understanding, genetic testing remains underused, even for those at high risk [26]. Current methods, like carbohydrate antigen 125 (CA-125) blood tests and transvaginal ultrasounds (TVUS) are not very accurate for catching early-stage disease [27]. CA-125 levels can rise due to non-cancerous conditions like endometriosis, menstruation, or pelvic inflammatory disease, causing false positives [27]. The United States Preventive Services Task Force (USPSTF) advises against screening asymptomatic women because of low early detection rates and frequent false positives [28]. While ultrasounds or magnetic resonance imaging (MRI) can spot masses, they usually cannot tell if they are benign or malignant without invasive biopsies. Promising new techniques, like human epididymis protein-4 (HE4) biomarkers, liquid biopsies, and multiomics, are being developed but are not ready for widespread use yet [29]. Advanced methods like liquid biopsies, artificial intelligence imaging analysis, and novel biomarker panels are still in early research phases [30].

Early-stage disease is typically silent, resulting in late diagnoses and lower survival rates. Non-specific symptoms often lead to ovarian issues being overlooked until later stages [31]. Symptoms like bloating, pelvic or abdominal pain, feeling full quickly, urinary urgency, constipation, and fatigue often resemble benign conditions like irritable bowel syndrome (IBS) or urinary tract infections (UTIs), causing misdiagnosis [31]. Tumors develop deep in the pelvic and abdominal cavity, making physical exams less effective for early detection [8]. Small ovarian masses are usually missed during routine pelvic exams [8]. Some subtypes, such as high-grade serous ovarian cancer (HGSOC), might originate in the fallopian tube rather than the ovarian surface, complicating detection further [32]. Because of rapid progression and late presentation, around 66–80% of ovarian cancers are diagnosed at stages III or IV, where the outlook is poor [27,33]. This limits the prognostic outcome of surgery and chemotherapy.

## 3. Impaired Immune Function in Peripheral Blood and Tumor Microenvironment of Ovarian Cancer Patients

Ovarian cancer presents major hurdles for immunotherapy and immune-based treatments due to its inherently immunosuppressive tumor microenvironment (TME) and patient-specific immune issues [34,35]. Many patients experience significant immune dysfunction in their peripheral blood, indicating systemic immune impairment in conjunction with local tumor-driven suppression [24,36]. In platinum-resistant recurrent ovarian cancer, hypoxia and fibrosis in the TME restrict immune cell trafficking and activation [37,38]. Additionally, many ovarian tumors display low neoantigen expression or tumor mutational burden (TMB), reducing immunogenicity and enabling immune evasion [39]. Advanced or recurrent ovarian cancer had increased myeloid-derived suppressor cells (MDSCs) in the peripheral blood [40]. Immunosuppressive niches, such as stromal cells, regulatory T cells (Treg), MDSCs, and tumor-associated macrophages (TAMs), weaken immune surveillance [41,42]. MDSCs and TAMs secreted immunosuppressive cytokines (IL-10, TGF-β) and metabolic enzymes, further blocking T-cell function [43,44,45]. FOXP3+ CD4+ Tregs suppressed effector T-cell responses via IL-10, TGF-β, the CD39/CD73 adenosine pathway, and IL-2 sequestration, while tumors attracted Tregs through CCL22/CCL28 to maintain immunosuppression [41,46,47]. Elevated peripheral Tregs contributed to resistance and disease progression [46].

Ovarian cancer patients showed a reduction in NK cell numbers and function in both peripheral blood and the TME [24,48,49]. Tumor-associated T and NK cells from ovarian carcinoma ascites exhibited defective signaling proteins, including reduced TcR-zeta chain and p56 (lck) expression, along with decreased IFN-γ, IL-2, and IL-4 gene expression [50,51,52,53]. CD16, an important FcγR for ADCC in NK cells, was unresponsive in tumor-associated NK cells, impairing ADCC against autologous tumor cells [24,54]. Studies on lymphocytes from the peritoneal cavity of ovarian cancer patients show reduced direct and ADCC cytotoxic activities, contributing to tumor growth and spread within the peritoneal cavity [55]. Surface antigen major histocompatibility complex-class I (MHC-class I) chain-related proteins A and B (MICA/B) mark them for elimination by NK cell-activating receptors via ADCC [23,56]. However, tumor cells, including epithelial ovarian cancer, can successfully evade detection by NK cells by proteolytic shedding of MICA/B from their surface [35,57]. Upon shedding, soluble MICA/B can downregulate NKG2D on NK cells, further impairing the anti-cancer activity of NK cells [57,58,59].

Tumor cells relied on glycolytic and glutamine metabolism, generating lactic acid, depleting nutrients, and acidifying plasma, which inhibited T-cell activation and proliferation [60,61]. Reduced intratumoral T cells were linked to increased vascular endothelial growth factor levels [62]. Chronic toll-like receptor-5 (TLR5) activation in dendritic cells led to weakened CD8+ T-cell activation and exhaustion in the TME, limiting the efficacy of checkpoint therapy [63]. CD8+ T cell exhaustion, identified by co-expression of inhibitory receptors like PD-1, TIM-3, LAG-3, TIGIT, and decreased cytotoxic markers like granzyme B and perforin, was observed in both tumor-infiltrating lymphocytes (TILs) and peripheral blood [64,65]. High levels of PD-1, TIM-3, and LAG-3 on peripheral CD8+ T cells suppressed immune responses, lowering immunotherapy effectiveness [66,67]. Defects in MHC-I molecules or antigen-presenting machinery further impede T cell recognition, contributing to primary resistance against ICIs [68]. Ovarian cancer patients often face impaired cytokine secretion from immune cells, resulting in poor tumor differentiation and a higher prevalence of ovarian CSCs, which drive tumor persistence, invasion, and metastasis [23,24,69]. Favorable cytokines like IFN-α2, IL-1β, IL-12p70, and MCP-1 are associated with improved survival, while elevated IL-6 and TNF-α levels correlate with worse outcomes, emphasizing the impact of systemic immune modulation reflected in serum cytokines [70,71,72].

These factors, mainly high Treg levels, CD8+ T cell exhaustion, reduced NK cell-mediated cytotoxicity, and altered peripheral cytokine profiles, make both conventional and new immunotherapeutic strategies less effective [73]. Developing novel cell-based immunotherapies and modulating the TME is essential for improving responses to immunotherapy and antibody-drug conjugates [34].

## 4. Tumor Heterogeneity in Ovarian Cancer

Ovarian cancer is known for being highly varied, both between different patients and within a single tumor itself. This disease is divided into type I tumors—endometrioid carcinoma (EC), clear cell carcinoma (CCC), low-grade serous carcinoma (LGSC), and mucinous carcinoma (MC)—and type II tumors, mainly high-grade serous carcinoma (HGSC) [74]. Type I tumors grow slowly with stable genomes, while type II tumors are aggressive, showing high *TP53* mutation rates and significant chromosomal instability [74]. Subtypes vary in therapy responses due to differences in DNA repair genes like *BRCA1/2*, *KRAS*, *PIK3CA*, and *TP53*, as well as the presence of diverse cancer cell populations within the same tumor, leading to resistance and inconsistent outcomes [75,76]. EC, often found at stage I, has a good prognosis, is linked to endometriosis, and commonly involves mutations in *CTNNB1*, *PIK3CA*, *ARID1A*, and *PTEN* [77]. CCC, with its glycogen-rich, clear cytoplasm, has a poor prognosis even when caught early, is sometimes connected to endometriosis, and frequently shows mutations in *PIK3CA*, *ARID1A*, and *PPP2R1A* [78]. LGSC, known for papillary structures and low mitotic activity, grows slowly but resists chemotherapy, with mutations in *KRAS*, *BRAF*, and *ERBB2*. MC, a rare subtype, is often diagnosed late, has poor outcomes, and features glandular, mucin-rich traits along with mutations in *KRAS*, *TP53*, and *ERBB2* [79]. HGSC, the most aggressive subtype, is typically found in advanced stages, characterized by papillary or solid architecture, nuclear atypia, widespread metastasis, and mutations in *TP53* and *BRCA1/2* [32]. Histologic subtypes are key in guiding therapy and predicting outcomes for ovarian cancer [74].

**Figure 1:**
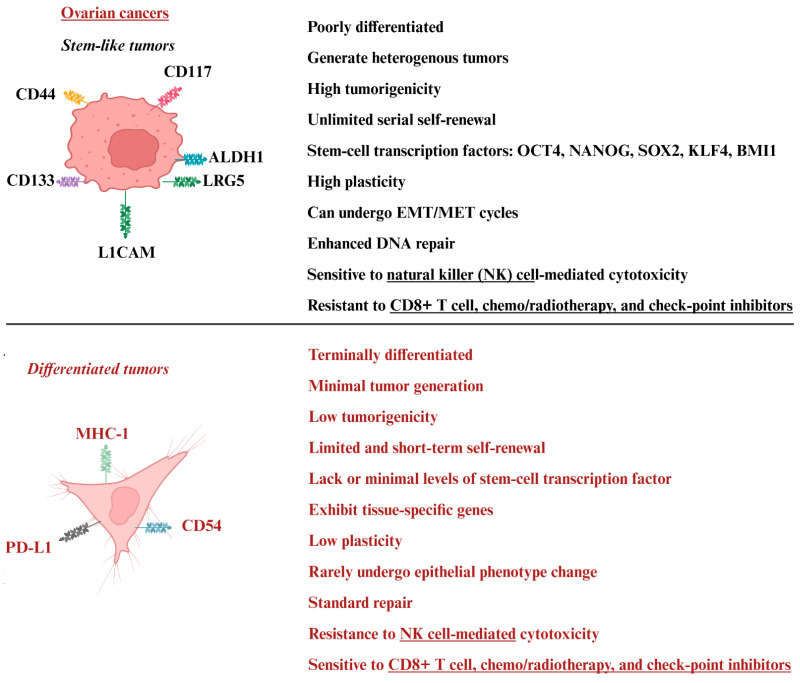
Illustration showing key characteristics differences between cancer stem cells (CSCs) and differentiated tumors. CSCs are poorly differentiated, expressing stem-like transcription factors, and are highly tumorigenic with unlimited self-renewal potential, whereas differentiated tumors are terminally differentiated, lacking stem-cell transcription factors and have limited or lack self-renewal potential. CSCs were found to be sensitive to NK cell-mediated cytotoxicity but mostly resistant to CD8+ T cells, chemotherapy, radiotherapy, and checkpoint inhibitors, whereas differentiated tumors were found to be resistant to conventional NK cell-mediated cytotoxicity but mostly sensitive to CD8+ T cells, chemotherapy, radiotherapy, and checkpoint inhibitors. Abbreviations: CD: cluster of differentiation; ALDH: aldehyde dehydrogenase; LRG5: leucine-rich repeat-containing G-protein-coupled receptor 5; L1CAM: L1 cell adhesion molecule; MHC-1: major histocompatibility class I; PD-L1: programmed cell death protein 1; OCT4: octamer-binding transcription factor 4; KLF4: krüppel-like factor 4; EMT/MET: epithelial-to-mesenchymal transition/mesenchymal–epithelial transition. Created in BioRender. Kaur, K. (2025) https://BioRender.com/xw7nbrp (accessed on 20 October 2025).

Most tumors are made up of a mix of different cell types, even when enriched with CSCs, and may still include a small group of moderately or well-differentiated cells. Ovarian tumors exhibit cellular diversity, often explained through the CSC model, alongside concepts of clonal evolution and plasticity [80,81]. Ovarian CSCs can self-renew, resist chemotherapy, and drive tumor growth, diversity, metastasis, and relapse [81]. Ovarian CSCs may arise from mutated ovarian stem/progenitor cells or through dedifferentiation of mature cells via epithelial–mesenchymal transition (EMT), metabolic changes, or cell fusion. Ovarian CSCs facilitate invasion and spread using EMT and mesenchymal–epithelial transition (MET), regulated by transcription factors like SNAIL, SLUG, ZEB1, and TWIST [81,82,83]. By symmetric or asymmetric division, they maintain stem-like properties and generate diverse cells that complicate the tumor structure [83] (Figure 1). Pathways such as TGF-β, Wnt, Notch, PI3K/AKT/mTOR, Hedgehog, NF-κB, and Hippo are vital for maintaining stemness [81]. Wnt/β-Catenin supports self-renewal and can be targeted with inhibitors like TF3, ginsenoside-Rb1, calcitriol, and sFRP4 [84]. The PI3K/AKT/mTOR pathway promotes survival and growth, with specific inhibitors available [85]. Other pathways, including Hedgehog, NF-κB, Hippo, TGF-β, and JAK/STAT, sustain ovarian CSCs and contribute to chemotherapy resistance [81].

The tumor microenvironment, consisting of stromal cells, cancer-associated fibroblasts, extracellular matrix, and hypoxic niches, supports CSCs, which thrive in low-oxygen conditions [81]. Their primary surface markers include CD44, CD133, CD24, CD117, LGR5, L1CAM, ALDH1, OCT4, NANOG, SOX2, and KLF4, though their expression varies based on tumor stage, heterogeneity, and microenvironment [23,86] (Figure 1). Drug-resistant ovarian CSCs, like CD44+CD117+ populations, help tumors survive chemotherapy [83]. These cells fuel tumor initiation, growth, and spread while resisting treatment through self-renewal and mechanisms such as enhanced DNA repair, anti-apoptotic signaling, dormancy, and drug-efflux transporters like ABCG2 [83]. More than 70% of advanced ovarian cancer patients relapse within two years of chemotherapy, largely due to CSCs [81]. Furthermore, they suppress immune responses by inducing M2 macrophage polarization, regulatory T cell expansion, and expressing CD24 to evade detection and clearance [87,88].

**Figure 2:**
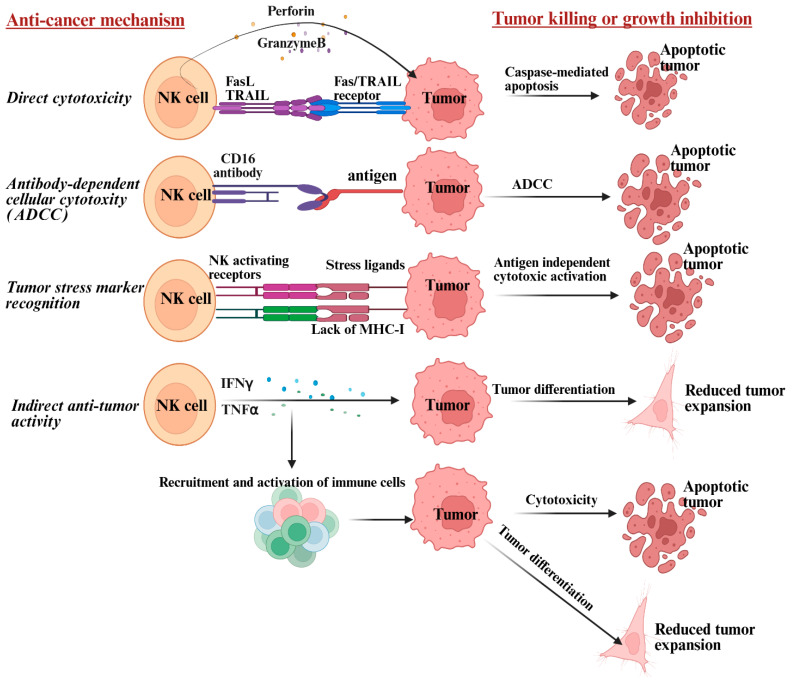
Illustration demonstrating NK cell-induced anticancer activity through direct cytotoxicity, ADCC, tumor recognition, activation of immune cells, and differentiation of tumors. NK cells take down tumor cells through the perforin/granzyme pathway, releasing perforin to punch holes in the tumor cell membrane and granzyme B to trigger caspase-driven apoptosis. They also carry out antibody-dependent cellular cytotoxicity (ADCC) when their receptors bind to the Fc portion of IgG antibodies attached to antigens on tumor cells, prompting granule release and cell lysis. Activating receptors on NK cells drives the cytotoxic response against tumors expressing ligands for those receptors. Cytokines from NK cells can affect tumor differentiation, reduce tumor growth, and make the tumors more sensitive to standard cancer treatments. Abbreviations: TRAIL: tumor necrosis factor (TNF)-related apoptosis-inducing ligand; CD: cluster of differentiation; MHC-1: major histocompatibility class I; IFN-γ: interferon-gamma. Created in BioRender. Kaur, K. (2025) https://BioRender.com/xfq9xu3 (accessed on 17 October 2025).

Differentiated tumors, unlike CSCs, have low tumorigenic potential, limited self-renewal, and reduced growth capacity [23] (Figure 1). As discussed in Section 5 and shown in Figure 2, NK cell-secreted cytokines such as IFN-γ and TNF-α can induce differentiation in CSCs, a process that limits tumor growth and reduces the chances of tumor recurrence [23]. Differentiated tumor cells show minimal levels of stem-cell transcription factors, tissue-specific gene expression, low plasticity, rare epithelial transitions, and lack efficient repair mechanisms (Figure 1) [82].

The success of immune cell-based cancer treatments often depends on the tumor’s characteristics. NK cells can tackle the complexity of ovarian tumors by targeting cells without relying on antigen presentation, making them especially effective in this setting. Their ability to bypass MHC restrictions and react to stress signals allows them to address tumor diversity. Supercharged NK cells, created using osteoclast feeder cells and probiotic bacteria, have been shown to attack both CSCs and differentiated tumors [24,89], showing their potential to target varied tumor types. While differentiated tumors respond better to chemo, radiation, and checkpoint inhibitors, CSCs are highly resistant to platinum-based therapies [90,91]. Figure 1 outlines the key differences between these two tumor types. Effective treatments should aim at eliminating CSCs to overcome drug resistance and prevent relapse or focus on targeting a wide range of tumor cells for complete eradication.

## 5. Mechanism by Which NK Cells Target Heterogeneous Tumor Populations and Limit Recurrence of Ovarian Cancer

Ovarian cancer is often called an immune “cold” tumor because it usually has low immune cell infiltration, weak antigen presentation, and a tumor environment that suppresses immune responses—factors that limit the success of standard immunotherapies [92]. High-grade serous ovarian cancer (HGSOC) in particular resists immune checkpoint inhibitors and other T cell-based treatments for several reasons [93]. These tumors often lack cytotoxic T lymphocytes (CTLs), which are key for adaptive immunity. They also downregulate MHC class I molecules and antigen-processing components, making it harder for T cells to recognize them [93]. NK cell-based therapies, however, can overcome these challenges because NK cells naturally recognize and kill tumor cells without needing antigen presentation or prior activation. As part of the innate immune system, NK cells can detect stress signals or missing “self” markers on cancer cells, offering a promising way to target immune “cold” tumors.

NK cells limit ovarian cancer recurrence through several mechanisms, including direct cytotoxicity, antibody-dependent cellular cytotoxicity (ADCC), cytokine secretion, immunological memory, and resistance to immune evasion [13,17,22]. They recognize and kill cancer cells when tumor cells downregulate major histocompatibility complex (MHC) class I molecules—a common immune evasion strategy [23]. Ovarian cancer stem cells (CSCs) were found to exhibit lower surface expression of MHC-class I, intercellular adhesion molecule 1 (ICAM-1 or CD54), and programmed death-ligand 1 (PD-L1) receptors, while exhibiting higher expression of CD44 [23]. NK cells have been shown to identify and attack ovarian CSCs expressing low MHC-class I via direct cytotoxicity, wherein NK cells induce direct cytotoxicity in tumor cells through the perforin/granzyme pathway, where they release perforin to create pores in the tumor cell membrane and granzyme B to trigger caspase-mediated apoptosis (Figure 2) [23,94,95]. NK cells also kill tumors via the death receptor pathway involving Fas ligand (FasL) or TRAIL binding to tumor cell receptors, activating caspase-8-mediated apoptosis [96].

NK cell receptors bind to antibodies coating tumor cells, triggering targeted killing, a process enhanced by monoclonal antibody therapies. ADCC in NK cells initiates when FcγRIIIA (CD16a) and/or FcγRIIC (CD32c) receptors on NK cells bind to the Fc portion of IgG antibodies (Figure 2). These IgG antibodies bind to antigens on the ovarian tumor cell surface, leading to granule exocytosis and tumor cell lysis [97]. FcγR CD16 and natural killer group 2 member D (NKG2D) were found as two major NK cell receptors for ADCC activation [97]. NK cells function by balancing activating signals that prompt them to target tumor cells with inhibitory signals that safeguard healthy cells [97,98] (Figure 2). NK cells activating receptors like NKG2D, DNAM-1, and NKp30/NKp44/NKp46 play a role in triggering cytotoxic response against tumors expressing ligands for these receptors [99]. Meanwhile, healthy cells display MHC-I molecules that bind to inhibitory receptors such as KIRs (Killer-cell Immunoglobulin-like Receptors) and NKG2A/CD94, preventing NK cells from attacking them [99].

NK cells also release IFN-γ and TNF-α, which inhibit tumor growth, recruit other immune cells, and make the tumor environment less suppressive. Cytokines secreted from NK cells play a crucial role in tumor differentiation (Figure 2) [100,101]. Differentiated tumors exhibit higher MHC-class I expression, whereas CSCs exhibit lower expression of surface MHC-class I [91,101,102]. Negative expression of MHC-class I in ovarian carcinoma was reported as one of the factors contributing to escape from the immune system [103,104,105,106,107,108]. Enhanced surface expression of MHC-class I in ovarian cancer also correlates with increased numbers of tumor-infiltrating lymphocytes, improved response to PD1/PDL1 therapy, better prognosis, and prolonged life of cancer patients [106,107,109,110]. Once differentiated, tumors express reduced growth and become sensitive to conventional cancer therapeutics. Research highlights a strong connection between tumor differentiation stages and their susceptibility to NK cell-mediated cytotoxicity, chemotherapy, radiation, CD8+ T cell therapies, and checkpoint inhibitors [91,101,111]. Therefore, NK cells play a key role in tumor differentiation, ultimately reducing the number of CSCs, leading to a reduction in tumor renewal, growth, spread, recurrence, and improved efficacy of conventional treatments [100,101].

These mechanisms of direct cytotoxicity, ADCC, tumor stress marker recognition, tumor differentiation, immunological memory, and resistance to immune evasion are indispensable for the effective targeting and reducing the chances of recurrence of tumor cells by the NK cells (Figure 2).

## 6. Main Differences Between T Cells and NK Cells in Targeting Ovarian Cancer

T cells, or T lymphocytes, are key players in the adaptive immune system [112]. They develop in the thymus and travel through the bloodstream, ready to detect and fight pathogens [112]. They are activated only when antigen-presenting cells (APCs) display specific antigens on MHC molecules [112]. Each T cell receptor (TCR) is unique to a single antigen, thanks to gene rearrangement during development [112]. Once activated, T cells can become memory cells, enabling a quicker, stronger response if the same antigen shows up again. Because activation requires antigen processing and presentation, the first response is slower, often taking a few days [113,114]. The main types are helper T cells (CD4+), cytotoxic T cells (CD8+), regulatory T cells, and memory T cells [112]. Helper T cells release cytokines to coordinate immunity, activate B cells, stimulate cytotoxic T cells, and recruit macrophages [115]. Cytotoxic T cells kill infected or cancerous cells by recognizing antigens on MHC class I molecules and releasing perforin and granzymes [116]. Regulatory T cells keep immune reactions in check, maintain tolerance to self-antigens, and help prevent autoimmunity, with defects often linked to autoimmune diseases [117]. Memory T cells provide long-term protection, responding rapidly to known threats, and can be central memory (long-lived in lymph nodes) or effector memory (fast responders in tissues) [113]. Ovarian CSCs typically exhibit reduced surface expression of MHC-class I, which may partly explain the limited success of T-cell-based immunotherapies in ovarian cancer patients [23,118]. Other subsets include naïve T cells [119], gamma-delta (γδ) T cells [120], and natural killer T (NKT) cells.

NK cells operate without needing antigens and are part of the innate immune system, while T cells depend on antigens and belong to the adaptive immune system [121,122]. They differ in their activation, specificity, and speed of response as listed in Table 1. Unlike T cells, NK cells do not need prior exposure to a specific antigen or MHC presentation to spring into action [123]. They detect stressed, infected, or abnormal cells by noticing the absence of “self” MHC class I molecules or the presence of stress-related ligands [124]. Earlier, it was believed that NK cells lack memory, but research now shows they can develop memory-like traits under certain conditions [125]. As mentioned in Section 2, NK cells kill targets directly with perforin and granzymes or shape immune responses by releasing cytokines like IFN-γ. Key differences between T cells and NK cells are listed in Table 1.

Understanding these differences is crucial in cancer immunotherapy as NK cells are harnessed for rapid tumor killing, while T cells (especially CAR-T cells) are engineered for targeted attacks. These differences suggest that NK cells may represent a valid alternative to T cells, due to their inherent nature as part of the innate immune response to aggressively attack MHC class I-deficient or mutated cells [21]. The effectiveness of NK cells in combating solid tumors is well-recognized, and ongoing studies emphasize the potential of NK cell-based therapies for ovarian cancer [23,24].

NKT cells, a hybrid of T cells and NK cells, exhibit combined traits of adaptive T cells and innate NK cells, carrying T-cell receptors like typical T cells but also NK markers such as NK1.1 and CD56 [130]. Unlike regular T cells, NKT cells respond to lipid antigens presented by CD1d molecules instead of peptide antigens via MHC-class I [131]. The most extensively studied group, Type I or invariant NKT (iNKT) cells, can release large amounts of IFN-γ to activate NK cells, dendritic cells, and cytotoxic T cells against tumors [132]. They also produce IL-4 and IL-10, which can reduce inflammation and, depending on the context, potentially weaken antitumor responses [132]. NKT cells can directly kill tumor cells or shape the tumor environment to strengthen immune defenses [133]. Researchers are exploring combination strategies that activate both NKT and NK cells, such as pairing CD1d agonists with NK cell infusions, to boost their cancer-fighting potential [134]. The key difference is that NK cells are innate lymphocytes without antigen-specific TCRs, while NKT cells are T cells with invariant TCRs that detect lipid antigens (Table 2). NK cells act as rapid killers, whereas NKT cells serve as versatile regulators bridging innate and adaptive immunity.

## 7. Current Ovarian Cancer Treatments and Their Limitations: How NK Cells Can Benefit

Among current treatments, cytoreductive surgery is a key treatment option for ovarian cancer, but often falls short in addressing microscopic metastases, leading to minimal residual disease (MRD) and recurrence [137]. Surgical stress can unintentionally trigger immunosuppression and angiogenesis, which support tumor growth [138]. Surgery is typically avoided for late-stage disease or patients with significant comorbidities, and achieving complete cytoreduction is especially difficult in stage III-IV cases [137,139]. Neoadjuvant chemotherapy helps shrink tumors before surgery, but its effectiveness varies, and extended cycles can reduce surgical benefits [140]. Chemotherapy, including platinum- and taxane-based drugs, is limited by its non-specific nature, causing systemic toxicity and side effects like myelosuppression, neuropathy, hair loss, and gastrointestinal issues [141,142]. Platinum-based treatments show high initial response rates (60–80%), but about 70% of patients relapse within three years [143]. Non-personalized dosing further reduces effectiveness while trying to balance tolerability [143]. Localized radiotherapy is less effective for metastatic or widespread peritoneal disease due to resistance from DNA repair, antioxidant pathways, and metabolic changes like GLUT1 overexpression [144,145]. Advanced techniques like intensity-modulated radiation therapy (IMRT) and stereotactic body radiation therapy (SBRT) minimize collateral damage but cannot fully overcome tumor resistance [146]. Hormonal therapies benefit hormone receptor–positive subtypes like low-grade serous ovarian carcinoma (LGSOC), though resistance develops through adaptations in the estrogen receptor/androgen receptor (ER/AR) pathways [147]. PARP inhibitors, such as Olaparib, Niraparib, and Rucaparib, are effective against BRCA-mutated or homologous recombination-deficient tumors but are less effective in homologous recombination (HR)-proficient tumors, where they may face resistance from restored homologous recombination or stabilized DNA replication forks [148,149]. Long-term use can cause rare but serious toxicities, with recent withdrawals of recurrent-use indications underscoring their narrow therapeutic range [149]. Anti-VEGF therapies like Bevacizumab are most effective for high-risk or chemo-resistant patients, improving progression-free survival (PFS) but offering minimal overall survival (OS) benefits for non-high-risk cases [150]. Resistance emerges from alternative angiogenic pathways, and side effects like hypertension, proteinuria, thrombosis, and bowel perforation are concerns [150]. These therapies may lose effectiveness in frontline use. Molecular profiling helps tailor treatments, such as PARP inhibitors for *BRCA* mutations or targeted inhibitors for *PIK3CA*, *PTEN*, or *ERBB2* alterations. However, prolonged therapies like chemotherapy, bevacizumab, and PARP inhibitors often lead to side effects, including myelosuppression, hypertension, and ocular toxicity [151].

Immune checkpoint inhibitors (ICIs) (PD-1/PD-L1, CTLA-4) are effective for tumors with high PD-L1 expression but face issues like an immunosuppressive tumor environment, low T-cell infiltration, and antigen variability [152,153]. In ovarian cancer, they show low objective response rates (ORR), influenced by tumor PD-L1 expression and the immunosuppressive tumor microenvironment [153]. Combining ICIs with PARP inhibitors or anti-angiogenics shows promise but adds toxicity risks [154]. Antibody-drug conjugates (ADCs) like Mirvetuximab soravtansine, targeting FRα, face challenges like off-target effects (keratitis, neuropathy, ILD) and limited use due to antigen heterogeneity, requiring biomarker-based stratification that restricts eligibility [155,156]. Novel small molecule inhibitors targeting *KRAS/MAPK*, *PI3K/AKT/mTOR*, *FAK*, and *ALK* have had limited success as monotherapies due to compensatory pathway activation [157,158]. Biomarker-guided combination therapies are being studied but remain in early-phase trials [159]. Comprehensive molecular profiling, like homologous recombination deficiency (HRD) testing and next-generation sequencing (NGS), along with biomarker selection, is essential but faces challenges such as tumor heterogeneity and high costs [160]. Progress continues, but personalized biomarker-based strategies and the complexity of multimodal care remain challenging. Oncolytic virotherapy (e.g., Olvi-Vec) aims to resensitize tumors to platinum, with phase III studies currently underway [161].

Combination therapies using chemotherapy, targeted therapy, immunotherapy, and ADCs need to address overlapping toxicities. Advancing early diagnostics, combination therapies, tumor environment targeting, and precision medicine is key to improving outcomes. Table 3 highlights the limitations of therapies discussed in this section, including the benefits NK cells could add based on preclinical and early clinical findings.

## 8. Challenges to Developing NK Cell-Based Immunotherapy

Since their discovery, biologists have worked to develop safe and effective strategies to treat cancer patients using NK cells [21,162]. While these cells are considered extremely safe for cell therapy, their efficacy has been a consistent challenge due to various issues. Unlike T cells, which make up 40–60% of lymphocytes, NK cells account for only 5–10% in peripheral blood [126] (Table 1). Additionally, NK cell function is compromised in cancer patients; for instance, ovarian cancer patients often exhibit reduced NK cell numbers and impaired functionality [24,48,49,54,55]. Patient-derived NK cells also show decreased activating receptor expression, reduced cytotoxicity, and lower IFN-γ secretion [24,53,163,164]. These factors hinder the effectiveness of ex vivo NK cell expansion protocols in achieving robust anti-cancer activity [164,165]. While cytokines can promote NK cell expansion, donor variability and the search for “super donors” remain significant obstacles [166]. Challenges like low transduction rates and inconsistent editing efficiency also limit gene editing and chimeric antigen receptors (CAR) engineering in NK cells [48,167,168]. Even with modifications like CAR-NK, achieving efficient gene transduction, stable expression, and identifying optimal tumor-specific antigens are ongoing hurdles [48,169]. Artificial feeder layer-dependent methods, such as K562, have shown limited success in maintaining long-term NK cell activation [165,170]. Scaling up potent NK cell populations ex vivo is challenging, and cryopreservation significantly reduces their viability and functionality after infusion.

Despite these difficulties, recent advancements have introduced methodologies to address inadequate ex vivo NK cell expansion. Efforts now focus on engineering NK cells with enhanced CAR constructs tailored to NK-specific needs.

## 9. Progress in NK Cell-Based Therapies for Ovarian Cancer: Insights from Preclinical Studies

**Figure 3:**
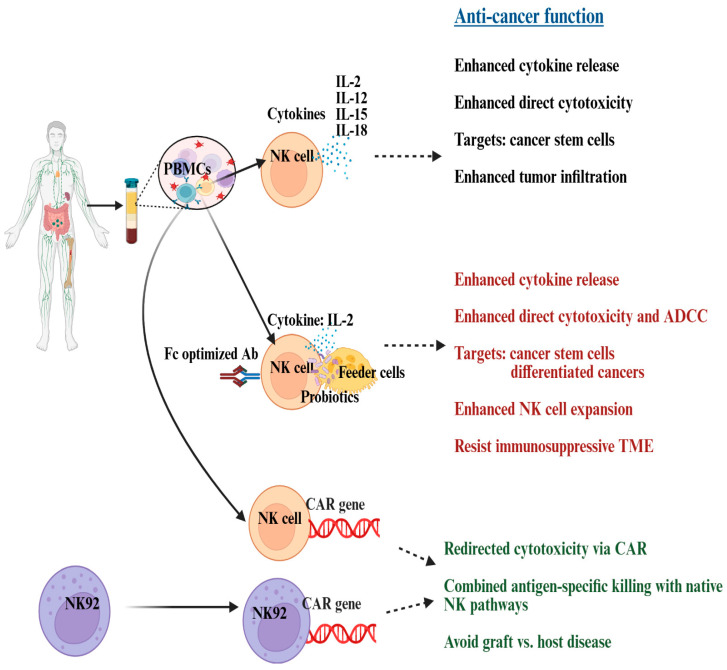
Illustration showcasing platforms for activating and expanding NK cells to develop NK cell-based immunotherapies. This can be achieved through cytokines (black text), a combination of feeder cells, cytokines, Fc antibodies, and probiotics (red text), or by utilizing CAR technology (green text). Abbreviations: PBMCs: peripheral blood mononuclear cells; NK: natural killer; IL: interleukin; ADCC: antibody-dependent cellular cytotoxicity; TME: tumor microenvironment; CAR: chimeric antigen receptor. Created in BioRender. Kaur, K. (2025) https://BioRender.com/rinjnt8 (accessed on 18 October 2025).

There is a strong link between NK cells’ ability to attack aggressive tumors, independent of MHC-class I expression [23]. The lower the MHC-class I levels on ovarian tumors, the better they respond to NK cells, except now a study on supercharged NK cells demonstrated that NK cells expanded using osteoclasts and probiotics could kill ovarian tumors irrespective of MHC-class I expression [23,24]. Preclinical NK cell therapies include cytokine-stimulated NK cells, memory-like NK cells, monoclonal antibody-activated NK cells, CAR-NK cells, and supercharged NK cells. These strategies have shown their efficacy in vitro and in vivo to lyse and inhibit the growth of ovarian cancer (Figure 3). This section highlights progress in NK cell-based therapies in preclinical ovarian cancer studies. 

Ex vivo activation of NK cells with cytokines like IL-2, IL-12, IL-15, or IL-18 enhances proliferation, cytokine secretion, and the ability to lyse ovarian cancer cell lines, even those with low MHC-I expression [167,171,172,173,174]. Cytokine activation, especially with IL-12, IL-15, and IL-18, boosts NK cell persistence, proliferation, and cytotoxic memory [48,173]. Cytokine-induced memory-like (CIML) NK cells show greater persistence and recall ability, providing long-term tumor surveillance [175,176]. Preclinical ovarian cancer models show CIML NK cells have superior tumor-killing abilities and persistence compared to conventional NK cells after adoptive transfer [48,173] (Figure 3). Fc-optimized monoclonal antibodies, such as B7-H3-targeted antibodies with S239D/I332E Fc modifications, improve CD16 binding and NK-mediated ADCC [177]. Preclinical studies demonstrate enhanced NK activation and increased IFN-γ and TNF-α release upon treatment with IL-2 and monoclonal antibodies against CD16. When the supernatant (culture media) of these NK cells was used for ovarian cancer cell lines, enhanced tumor differentiation was seen [23].

Supercharged NK cells, created using osteoclasts as feeder cells and probiotic bacteria, show higher cytotoxic receptor expression, increased IFN-γ and TNF-α secretion, and robust lysis of both stem-like and differentiated ovarian cancers, irrespective of MHC-I expression [24] (Figure 3). Targeting ovarian tumors irrespective of MHC-class I expression on tumor cells makes them a unique NK cell-based treatment strategy for effective targeting of heterogeneous solid tumors such as ovarian cancers [24]. Enhanced IFN-γ and TNF-α cytokine release from supercharged NK cells promotes tumor differentiation by increasing MHC-class I surface levels in stem-like ovarian tumors [24]. Enhancing MHC-class I expression in these tumors could enable T cells or chemotherapeutic drugs to target them effectively. When supercharged NK cells were infused into humanized mice (healthy and solid cancer-bearing) and observed for eight weeks, no sign of toxicity, pain, distress, or adverse events as cytokine-release syndrome (CRS) or immune effector cell-associated neurotoxicity syndrome (ICANS), was detected [178,179]. Supercharged NK cells can bypass tumor resistance mechanisms and remain effective even in suppressive conditions [24,178]. When supercharged NK cells were adoptively transferred into humanized mice with solid tumors (oral, pancreatic, and melanoma), they notably slowed tumor growth, encouraged in vivo tumor differentiation, boosted peripheral NK cell function, enhanced immune cell infiltration into the tumor microenvironment, and increased CD8+ T cell levels in both tumor and peripheral tissues [102,165,170,178,179,180,181]. These findings and positive findings from ovarian cancer in vitro studies suggest promising outcomes for ovarian cancer-bearing humanized mice, with related studies currently underway.

Preclinical models indicate that adoptive transfer of activated NK cells can shrink tumors and delay recurrence, and ongoing research is exploring combinations with checkpoint inhibitors, cytokines, and monoclonal antibodies to boost efficacy and durability [25]. NK cells, when adoptively transferred in preclinical studies, have interacted with dendritic cells in the tumor microenvironment, attained a memory-like phenotype, and maintained cytotoxicity against the tumor [25]. A cell-free approach has been explored using exosomes from expanded NK cells, and they were found to exhibit cytotoxic and immunomodulating properties to improve the delivery and efficacy of chemo-drugs in ovarian cancer [182].

Chimeric antigen receptor (CAR) engineering equips NK cells with synthetic receptors that can recognize specific tumor antigens, enhancing their precision and effectiveness. CAR-NK cells have shown the ability to overcome resistance and improve tumor targeting, particularly in epithelial ovarian cancer (EOC). Current research focuses on developing universal, safe, and potent CAR-NK or CAR-NKT therapies to address the limitations of CAR-T in solid tumors [183]. Allogeneic CAR-NKT cells derived from hematopoietic stem cells overcame ovarian cancer’s resistance to standard CAR-T therapy, achieving stronger antitumor effects with a favorable safety profile [183]. Compared to CAR-T cells, CAR NK cells may offer lower toxicity, ready-to-use availability, and improved safety [48]. Gene-edited CAR NK cells are under investigation for advanced ovarian cancer, though challenges persist in producing sufficient cells, boosting their tumor-homing ability, and ensuring their survival in hostile tumor environments. Memory-like CAR NK cells targeting the membrane-proximal domain of mesothelin—a protein abundant in ovarian cancer—have delivered promising results in preclinical studies [184]. CAR-NK cells targeting ovarian cancer antigens and altering the tumor microenvironment have successfully eradicated resistant ovarian tumors, including high-grade serous ovarian cancer, with sustained cytotoxicity and improved infiltration (Figure 3) [172,184,185,186,187,188,189,190].

## 10. Progress in NK Cell-Based Therapies for Ovarian Cancer: Insights from Clinical Studies

To tackle aggressive ovarian tumors, it is crucial to restore and enhance NK cell function in patients. Tumors with CSCs and low MHC-class I levels require NK cell-based therapies to either destroy or differentiate them [23]. Besides NK cells directly targeting these tumors, differentiation makes them more susceptible to chemotherapy, radiotherapy, checkpoint inhibitors, and T cell-driven cytotoxicity [23] (Figure 2). Combining NK cells with traditional therapies is key to successfully treating these tumors. Studies show that allogeneic NK cells are readily available and do not require generation from a patient’s own cells [191]. Additionally, NK cell therapy has not been linked to GVHD or cytokine storms [178,179]. These advantages position NK cells as an excellent option for tumor immunotherapy. This section highlights progress in NK cell-based therapies in clinical ovarian cancer studies (Table 4). Current studies are exploring the safety, tolerability, and cancer-fighting potential of memory-like NK cells in ovarian cancer patients, striving to boost anti-tumor immune responses in recurrent cases. 

Cytokine-induced memory-like (CIML) NK cells exhibit memory-like responses, boosting NK cells’ ability to detect and destroy cancer cells. In ovarian cancer, NK cells stimulated with IL-12, IL-15, and IL-18, known as CIML NK cells, have shown promising clinical trial results [48,175]. Research continues to enhance NK cell effectiveness and durability, with trials focusing on memory-like and CIML NK therapies for recurrent or platinum-resistant ovarian cancer [184]. These approaches aim to improve NK cell persistence, anti-tumor activity, and patient outcomes, with early findings indicating safety and potential for disease stabilization [167]. Ex vivo cytokine induction with IL-12, IL-15, and IL-18 creates a “memory-like” phenotype, enhancing proliferation, persistence, and tumor recognition [175]. A Phase 1B trial (ClinicalTrials.gov identifier: NCT06321484) is testing patient-derived CIML memory-like NK cells (IL-12, IL-15, IL-18) with chemotherapy via intraperitoneal infusions; results have shown improved antitumor activity and persistence compared to conventional NK cells. Another study combining CIML with IL-2 support has shown benefits for ovarian cancer patients, with ongoing research evaluating adverse events and clinical responses [192].

Geller et al. (ClinicalTrials.gov identifier: NCT01105650) treated 14 ovarian cancer patients with haploidentical IL-2-activated NK cells followed by subcutaneous IL-2 infusions three times weekly for 14 days, detecting NK cell expansion in their peripheral blood [193]. Xie et al. reported a case of an ovarian cancer patient receiving ex vivo expanded NK cells every two weeks for six total infusions, resulting in significant tumor mass reduction and prolonged survival with minimal side effects [194]. Of the 14 patients in clinical trial NCT01105650, six experienced progression-free survival at one year.

CAR-NK or CAR-NKT cells are emerging as a promising immunotherapy for ovarian cancer, showing encouraging early clinical results. Trophoblast cell-surface antigen 2 (TROP2)-CAR-NK cells engineered with IL-15 and delivered intraperitoneally are under study for platinum-resistant ovarian cancer [195] (ClinicalTrials.gov identifier: NCT05922930). NiKang Therapeutics (ClinicalTrials.gov identifier: NCT06586957) has started trials in solid cancers, including ovarian cancer, to evaluate the safety, tolerability, pharmacokinetics, and preliminary anti-tumor activity of NKT cells.

Clinical trials indicate that NK cell therapy is generally well-tolerated, with many patients experiencing disease stabilization, though response rates can differ [48]. These studies represent significant progress in developing new immunotherapies for ovarian cancer, offering hope for improved outcomes in patients with recurrent or platinum-resistant tumors. The future of NK cell-based treatments looks promising, with ongoing research aimed at enhancing their effectiveness. However, high-quality, large-scale data are still limited, emphasizing the need for continued trials to confirm effectiveness and integrate NK cell therapies into standard ovarian cancer treatment.

## 11. Challenges and Limitations of NK Cell-Based Therapies for Ovarian Cancer

Adoptively transferred NK cells often face challenges with survival and proliferation in vivo, particularly within the immunosuppressive tumor microenvironment (TME) [25,167,196]. Physical barriers and low chemokine levels further impede NK cell recruitment and infiltration into ovarian cancer [48,196]. While cytokine therapies like IL-2 and IL-15 can activate NK cells, they often result in severe toxicities, including vascular leak syndrome and cytokine release syndrome [48,197]. Tumors can evade NK cell function by downregulating activating ligands or releasing soluble ligands like MICA/B, which engage inhibitory NK receptors and cause dysfunction [196,198,199,200]. Tumors may also evade NK cell-mediated killing by maintaining or increasing MHC class I molecule expression, activating inhibitory KIRs on NK cells [34,48]. Inhibitory checkpoint molecules such as NKG2A, TIGIT, and PD-1 contribute to NK cell exhaustion and reduced cytotoxicity [201,202,203]. Additionally, NK cells are quickly inactivated in the TME [204]. Factors such as TGF-β, IL-10, hypoxia, elevated adenosine, reactive oxygen species, prostaglandins, and suppressive cells like Tregs, MDSCs, and TAMs further suppress NK cell survival and activation [41,42,205,206,207]. To tackle these challenges, advanced strategies focus on creating memory-like NK cells, including cytokine-induced memory-like NK cells, to improve their persistence and antitumor effectiveness (Table 5).

Allogeneic NK cells are promising for adoptive therapies due to their lack of GVHD risk, but they face challenges like immune rejection and limited persistence from HLA mismatches [208,209]. A key issue is that most recipients have functional immune systems that recognize and reject these foreign cells [11]. Host T cells may attack donor cells with mismatched HLA, B cells can create alloantibodies tagging NK cells for destruction, and host NK cells might target donor cells lacking self-HLA ligands [210,211]. Macrophages and complement activation also contribute to eliminating donor NK cells [212]. This rejection reduces the persistence and effectiveness of NK cell therapies [213]. Selecting donors based on KIR and HLA compatibility can enhance NK cell function and reduce rejection, while repeated dosing of off-the-shelf NK products could improve therapeutic outcomes [214].

**Table 5:** Major challenges of NK cell-based therapy, along with potential solutions and combination strategies.

Challenge/Limitation	Potential Solutions: Developing NK Cell-Based Therapies	Strategies for Therapeutic Benefits	References
Limited survival and proliferation in vivo	- Genetic engineering (e.g., CAR, IL-15 transgene) -Cytokine support (IL-2, IL-15, IL-21) to generate memory like NK cells - Supercharging using osteoclasts as feeder cells combined with probiotics	Combine with cytokine therapy or checkpoint inhibitors to sustain activity	[25,167,169,196,215]
Poor trafficking and infiltration into solid tumors: due to physical barriers and low chemokine levels	- Chemokine receptor engineering (e.g., CXCR4, CCR7) - Preconditioning regimens to remodel stroma	Combine with oncolytic viruses or stromal-targeting drugs to enhance infiltration	[48,196,216,217]
Immunosuppressive tumor microenvironment (TME): Tumors secrete TGF-β, IL-10, and express inhibitory ligands that suppress NK function	- Blockade of inhibitory pathways (e.g., anti-TGF-β, anti-PD-1/PD-L1) - Metabolic reprogramming - Cytokine support (IL-2, IL-15, IL-21) to generate memory like NK cells - Supercharging using osteoclasts as feeder cells combined with probiotics	Combine NK therapy with immune checkpoint inhibitors or metabolic modulators	[41,42,169,201,202,203,204,205,206,207,216,218].
Tumors can evade NK cells by releasing soluble ligands or by upregulating MHC-class I: soluble ligands can further inhibit NK cells’ anti-cancer activity	- Supercharging using osteoclasts as feeder cells combined with probiotics - Cytokine support (IL-2, IL-15, IL-21) to generate memory like NK cells	-Modification of TME -Personalized approaches of tailoring NK cell therapies based on tumor immunogenicity	[196,198,199,200].
Limited tumor specificity: NK cells rely on natural cytotoxicity, which may not be sufficient for heterogeneous tumors	- CAR-NK cells engineered with tumor-specific receptors - Supercharging using osteoclasts as feeder cells combined with probiotics	- Combine CAR-NK with monoclonal antibodies (ADCC) or bispecific engagers -Modification of TME -Personalized approach	[215,216]
Manufacturing and scalability issues: Difficulty in producing large, standardized NK cell products	- Use of induced pluripotent stem cells (iPSCs), cord blood, or NK cell lines as “off-the-shelf” sources - Supercharging using osteoclasts as feeder cells combined with probiotics	- Combine with universal donor platforms and cryopreservation technologies	[215]
Risk of limited efficacy compared to CAR-T: NK cells show lower persistence and potency in some cancers	- Optimize CAR constructs, enhance signaling domains, and improve expansion protocols	- Combine CAR-NK with CAR-T or other immune therapies for synergistic effects	[215,216,217]
Potential toxicity and safety concerns: Vascular leak syndrome and cytokine release syndrome (CRS) are less common but possible.	- Careful dose escalation, suicide gene switches, safety switches in engineered NK cells - Supercharging using osteoclasts as feeder cells combined with probiotics	- Combine with supportive therapies to mitigate toxicity	[48,169,197,215]
Allogenic NK cells: Immune rejection and limited persistence from HLA mismatches	- Selecting donors based on KIR and HLA compatibility	-Repeated dosing	[208,209,210,211]

Abbreviations: CAR: chimeric antigen receptor; IL: interleukin; NK: natural killer; CXCR4: C-X-C chemokine receptor type 4; CCR7: C-C chemokine receptor type 7; TGF-β: transforming growth factor beta; PD1: programmed cell death protein 1: PD-L1: programmed death ligand 1; ADCC; antibody dependent cellular cytotoxicity; KIR: killer-cell immunoglobulin-like receptors; HLA: human leukocyte antigen.

## 12. Exploring Future Strategies to Enhance the Effectiveness of NK Cell-Based Therapies for Ovarian Cancer

In resistant ovarian cancers, combining single-cell profiling, modifying the tumor microenvironment, nanocarrier delivery, and combination therapies can boost NK cell therapy by improving tumor targeting, overcoming immune evasion, and enhancing persistence (Table 5). Single-cell RNA sequencing and proteomics help map NK cell diversity, uncovering subsets with higher cytotoxicity or signs of exhaustion [127]. This paves the way for personalized treatments by pinpointing patient-specific NK cell issues, like inhibitory receptor overexpression or metabolic stress, that can be addressed before infusion [127]. CRISPR-based screens in NK cells reveal key regulators (such as MED12, ARIH2, CCNC) that, when tweaked, boost NK cell killing power [219]. This approach gives ovarian cancer patients access to selected or engineered NK subsets—like adaptive or memory NK cells—with better persistence and tumor recognition. Ultimately, single-cell insights make it possible to tailor NK therapy to each patient’s tumor biology for maximum impact. Personalized approaches to tailoring NK cell therapies based on tumor immunogenicity and patient-specific factors may enhance outcomes. Efforts to enhance NK cell efficiency include modifying the tumor microenvironment, aiming to improve outcomes and survival rates for ovarian cancer patients.

Nanocarriers can deliver cytokines like IL-15 and IL-21, checkpoint inhibitors, or gene-editing tools directly to NK cells or the tumor microenvironment [220]. By focusing delivery at the local site, they reduce systemic side effects compared to free cytokines or drugs. These carriers can be engineered to pass through ovarian tumor stroma and release activating agents that improve NK cell infiltration. This approach also allows for delivering siRNA or CRISPR components to disable inhibitory pathways in NK cells, enhancing their resistance to suppressive tumor signals [221,222].

Future research should explore combining NK cell-based therapies with immune checkpoint inhibitors, CAR-T, PARP inhibitors, or anti-angiogenic drugs to counteract the immunosuppressive tumor microenvironment, overcome resistance, and to reduce recurrence rates. New monoclonal antibodies like Siglec-7 glyco-immune binding MAbs attract NK cells to tumors, boosting their cytotoxic activity. NK engagers and nanocarrier targeting help minimize off-target effects, focusing the attack on resistant cancer cells. Memory-like NK cells provide stronger recall responses to tumor antigens, offering long-term surveillance. Blocking immunosuppressive cytokines such as TGF-β can revive NK activity in resistant cancers, while adaptive NK cells supported by cytokines can survive longer in the challenging tumor environment. Chemotherapy preconditioning may enhance NK cell homing and persistence, while experimental approaches like ligand-targeted delivery and catheter-based methods deserve further investigation. Additionally, studying probiotic supplementation and identifying beneficial strains as adjuvant therapies for ovarian cancer is a promising area.

Single-cell profiling maps the plan, nanocarriers provide the tools, TME modification and combination immunotherapy add the support. Together, they make NK therapy a more precise, durable, and powerful way to take on stubborn ovarian cancers.

Unanswered questions remain, such as whether activators beyond IFN-γ and TNF-α can upregulate MHC-class I in ovarian CSCs or induce their differentiation. Furthermore, could receptors like MHC class II and MHC class I play a role in the therapeutic targeting of ovarian cancer?

## 13. Conclusions

This review highlights how adaptive NK cells can recognize and target ovarian tumors, paving the way for innovative immunotherapy. While conventional treatments for ovarian cancer have made progress, side effects, toxicity, and long treatment cycles still impact patients’ quality of life and adherence, and the majority of cases show tumor recurrence. NK cell-based therapies are advancing rapidly from preclinical research to clinical trials, with memory-like and adaptive NK cells leading the way. Preclinical NK cell therapies include cytokine-stimulated NK cells, memory-like NK cells, monoclonal antibody-activated NK cells, CAR-NK cells, and supercharged NK cells. Positive results from a preclinical study encourage clinical studies. Ongoing clinical trials aim to evaluate their safety, effectiveness, and compatibility with current treatments. Although NK cell-based therapies were shown to be very promising, there are still challenges, especially NK cells’ survival and expansion in vivo and retained function in complex TME. Looking ahead, promising future strategies can be personalized therapy, well-equipped delivery tools, engineering NK cells alone or in combination therapies, targeted therapies, and microenvironment modification to enhance treatment effectiveness and improve survival rates for patients with advanced or resistant ovarian cancer.

## Figures and Tables

**Table 1:** Key differences between antigen-dependent T cell responses and antigen-independent NK cell activity.

Characteristics	T Cells	Natural killer (NK) Cells	References
Percentages in human peripheral blood mononuclear cells (PBMCs)	40–70%	5–20%	[126]
Major subsets	Helper T cells (CD4+), Cytotoxic T cells (CD8+), Regulatory T cells	Single main type, but with activating and inhibitory receptors	[112,127]
Immune System Role	Adaptive immunity	Innate immunity	[112,123]
Response Time	Slower (days)	Rapid (hours)	[112,123]
Activation	Require antigen presentation via MHC molecules	Do not require prior exposure; detect absence of MHC class I or presence of stress signals	[112,124]
Specificity	Highly specific to particular antigens	Broad recognition of stressed, infected, or tumor cells	[112,124]
Memory	Generate long-term immunological memory	Limited or conditional	[113,125]
Risks of graft vs. host disease in an allogeneic setting	Higher	Lower	[128,129]

Abbreviations: MHC-1: major histocompatibility class I; CD: cluster of differentiation.

**Table 2:** Key differences between conventional NK and NKT cells.

Characteristics	Natural killer (NK) Cells	Natural killer T (NKT) Cells	References
Lymphocyte type	Large granular lymphocytes (innate immune cells)	Specialized T lymphocytes (subset of T cells)	[135]
Percentage in human peripheral blood mononuclear cells (PBMCs)	5–20%	Rare in blood (<1%), enriched in liver and thymus	[136]
Gene rearrangement	Do not rearrange TCR genes	Rearrange TCR genes	[136]
Key surface marker	CD56 and CD16; lack CD3	invariant αβ TCR/CD3	[136]
Antigen Recognition	Recognize stress ligands and missing-self signals via activating/inhibitory receptors	Recognize lipid antigens presented by CD1d molecules	[133]
Immunity Role	Part of innate immunity; rapid, non-specific cytotoxic response	Bridge innate and adaptive immunity; can rapidly produce cytokines and influence adaptive responses	[130]
Cytokine Production	Produce IFN-γ, TNF-α; promote Th1 responses	Produce large amounts of IL-4, IFN-γ, and other cytokines; modulate both Th1 and Th2 responses	[130,132]
Memory	No classical memory; short-lived “fight or flight” cells	Some memory-like features due to TCR-mediated activation	[134]

Abbreviations: TCR: T cell receptor; CD: cluster of differentiation; IFN-γ: interferon-gamma; TNF-α: tumor necrosis factor-alpha; Th: T helper.

**Table 3:** Current Ovarian Cancer Treatments and their Limitations: How NK cells can benefit.

Therapy Type	Limitations	How May Natural Killer (NK) Cell Therapy Benefit?	References
Cytoreductive surgery	- Falls short in addressing microscopic metastases - High recurrence rate - Can trigger immunosuppression and angiogenesis, supporting tumor growth - Procedure is challenging and has limited efficacy in advanced disease	- NK cells target CSCs, reducing the chances of recurrence - Secreted cytokines support the immune system - Tumor differentiation reduces metastasis and recurrence	[137,138,139]
Surgery + Chemotherapy	- High recurrence rates due to drug resistance - Severe side effects (nausea, neuropathy, bone marrow suppression) - Limited efficacy in advanced/recurrent disease - Non-personalized dosing further reduces effectiveness	- Direct killing of resistant tumor cells without relying on DNA damage pathways, potentially reducing recurrence - Minimal or no severe side effects	[140,141,142,143]
Localized radiotherapy	- Limited efficacy in advanced/recurrent disease	- Direct killing of resistant tumor cells without relying on DNA damage pathways, potentially reducing recurrence	[144,145,146].
Hormonal therapies	- Resistance develops through adaptations	- Reduced chances of recurrence by targeting CSCs - Tumor differentiation reduces metastasis and recurrence	[147]
Targeted Therapy (PARP inhibitors)	- Works mainly in BRCA-mutated or HR-deficient tumors - Resistance develops over time - Not effective for all patients - Long-term use can cause rare but serious toxicities	- NK cells act independently of BRCA/HR status, broadening applicability across patient groups	[148,149]
Anti-VEGF therapies	- Limited overall survival (OS) benefits for non-high-risk cases - Resistance emerges from alternative angiogenic pathways - Benefits vary by tumor subtype - Can worsen prognosis in some immune-infiltrated tumors - Risk of hypertension, bleeding, thrombosis, proteinuria, and bowel perforation	- NK cells bypass angiogenesis pathways, directly attacking tumor cells and modulating the immune microenvironment	[150,151]
Immune checkpoint inhibitors	- Limited response rates in ovarian cancer due to the immunosuppressive tumor environment - Tumor immune evasion mechanisms reduce efficacy	- NK cells recognize tumors without antigen priming, overcoming immune evasion and low mutational burden	[152,153]
Immune checkpoint inhibitors + PARP inhibitors	- Toxicity risks	- Minimal or no severe side effects	[154]
Antibody-drug conjugates	- Off-target effects (keratitis, neuropathy, interstitial lung disease) - Limited use due to antigen heterogeneity - Require biomarker-based stratification that restricts eligibility	- Can target heterogeneous tumor population - No biomarker-based stratification is needed	[155,156]
Experimental Therapies	- Early-stage, limited clinical data - May face delivery and durability challenges	- NK cells (especially “memory-like” or adaptive NK cells) show persistence and recall ability, enhancing long-term tumor control	[157,158].

Abbreviations: CSCs: cancer stem cells; DNA: deoxyribonucleic acid; BRCA: breast cancer gene; HR: homologous recombination; PARP: Poly (ADP-ribose) polymerase.

**Table 4:** Clinical trials investigating NK cell-based therapies for ovarian cancer, both ongoing and completed.

NK Cell Activation	Trial Phase	ClinicalTrials.gov Identifier	Patients Enrolled
Allogeneic CD3/CD19 depleted NK+ IL-2	Completed	NCT01105650	13
Allogeneic NK+ IL-12	Terminated	NCT00652899	12
Haploidentical NK+IL-2 + indoleamine-2,3-dioxygenase	Completed	NCT02118285	2
Allogeneic NK + IL-2	Completed	NCT03213964	10
Cryosurgery + NK	Completed	NCT02849353	30
TROP2-CAR IL-15 transduced cord blood-NK	Phase 2	NCT05922930	51
Anti-mesothelin CAR-NK	Phase 1	NCT03692637	30
Autologous activated NK	Phase 2	NCT03634501	200
Ex vivo-generated UCB-derived allogeneic NK + IL-2	Completed	NCT03539406	11
Cytokine-induced NK cells + Radiofrequency ablation	Phase 2	NCT02487693	50
Cytokine (IL-2)-induced memory-like NK	Phase 1	NCT06321484	18
NKG2D CAR-NK	Unknown	NCT05776355	18
NKT	Phase 1	NCT06586957	150

Abbreviations: CD: cluster of differentiation; NK: natural killer; IL: interleukin; TROP2: trophoblastcell-surfaceantigen2; CAR: chimeric antigen receptor; UCB: umbilical cord blood; NKG2D: natural killer group 2 D; NKT: natural killer T.

## Data Availability

No new data were created or analyzed in this study.

## Abbreviations

The following abbreviations are used in this manuscript:

ICI	Immune checkpoint inhibitors
PARP	Poly (ADP-ribose) polymerase
CSCs	Cancer stem cells
NK	Natural killer
IFN-γ	Interferon-gamma
TNF-α	Tumor necrosis factor-alpha
CAR	Chimeric antigen receptor
CD	C-Reactive protein
NKG2D	Natural killer group 2D
CD	Cluster of differentiation
IL	Interleukin
ADCC	Antibody-dependent cellular cytotoxicity
PD-L1	Programmed death ligand-1
MHC-I	Major histocompatibility complex-class I
MICA/B	MHC class I polypeptide-related sequence A and B
MDSCs	Myeloid-derived suppressor cells
TAM	Tumor-associated macrophages
TME	Tumor microenvironment
Treg	Regulatory T cells
TIL	Tumor-infiltrating lymphocytes
KIR	Killer-cell immunoglobulin-like receptors
HLA	Human leucocyte antigen
